# Examining Wing Length–Abundance Relationships and Pyrethroid Resistance Mutations among *Aedes albopictus* in a Rapidly Growing Urban Area with Implications for Mosquito Surveillance and Control

**DOI:** 10.3390/ijerph18189443

**Published:** 2021-09-07

**Authors:** Stephanie J. Mundis, Gabriela Hamerlinck, Emily K. Stone, Ari Whiteman, Eric Delmelle, Tyler Rapp, Michael Dulin, Sadie J. Ryan

**Affiliations:** 1 Quantitative Disease Ecology and Conservation Lab, Department of Geography, University of Florida, Gainesville, FL 32611, USA; ghamerlinck@ufl.edu (G.H.); emily.stone@ufl.edu (E.K.S.); 2 Emerging Pathogens Institute, University of Florida, Gainesville, FL 32611, USA; 3 Department of Geography and Earth Sciences and Center for Applied Geographic Information Science, University of North Carolina at Charlotte, Charlotte, NC 28223, USA; osa0@cdc.gov (A.W.); Eric.Delmelle@uncc.edu (E.D.); tyler_rapp@bellsouth.net (T.R.); 4 Academy Population Health Initiative, Charlotte, NC 28223, USA; mdulin3@uncc.edu; 5 School of Life Sciences, University of KwaZulu-Natal, Private Bag X54001, Durban 4000, South Africa

**Keywords:** *Aedes albopictus*, pyrethroid resistance, morphological traits, weather cues

## Abstract

*Aedes albopictus* is a cosmopolitan mosquito species capable of transmitting arboviruses such as dengue, chikungunya, and Zika. To control this and similar species, public and private entities often rely on pyrethroid insecticides. In this study, we screened *Ae. albopictus* collected from June to August 2017 in Mecklenburg County, a rapidly growing urban area of North Carolina, for mutations conferring pyrethroid resistance and examined spatiotemporal patterns of specimen size as measured by wing length, hypothesizing that size variation could be closely linked to local abundance, making this easily measured trait a useful surveillance proxy. The genetic screening results indicated that pyrethroid resistance alleles are not present in this population, meaning that this population is likely to be susceptible to this commonly used insecticide class. We detected no significant associations between size and abundance-related factors, indicating that wing-size is not a useful proxy for abundance, and thus not useful to surveillance in this capacity. However, mosquitoes collected in June were significantly larger than July or August, which may result from meteorological conditions, suggesting that short-term weather cues may modulate morphological traits, which could then affect local fecundity and virus transmission dynamics, as previously reported.

## 1. Introduction

Recent emergences and spread of diseases such as dengue, chikungunya, and Zika have led to an uptick in public interest and concern about vector control for public health in the United States. Greater knowledge of the presence and distribution of disease vectoring *Aedes* mosquitoes can inform vector control. However, an additional factor has emerged in recent years: insecticide resistance. This study undertook an examination of vector surveillance data for *Aedes albopictus* mosquitoes in Mecklenburg County, a rapidly growing urban area in the state of North Carolina, to assess potential factors affecting distribution and evidence for the emergence of insecticide resistance.

First identified in the state of Texas in 1985, the invasive *Ae. albopictus* has dramatically expanded its range in the United States [1,2]. This expansion is part of a global trend: in the last 50 years, *Ae. albopictus* has spread to all inhabited continents [3] and has become established in both tropical and temperate environments [1]. This species is a container-breeder and feeds opportunistically, biting a wide range of hosts, although some populations exhibit a preference for mammals, and, more specifically, humans [4]. While *Ae. albopictus* is less anthropophilic than *Ae. aegypti*, it can serve as a vector for the same arboviruses as *Ae. aegypti* including dengue, chikungunya, Rift Valley fever, yellow fever, and Zika viruses [5]. Moreover, the opportunistic feeding behavior of *Ae. albopictus* may allow this species to act as a bridge vector, leading to spillover of zoonotic pathogens into human populations [4]. Additionally, populations of *Ae. albopictus* often competitively displace populations of *Ae. aegypti* [6].

Given the vector status of *Ae. albopictus*, its recent global expansion, and its ability to out-compete other important vector species, it is unsurprising that this species has affected public health and been accordingly targeted by vector control programs. *Ae. albopictus* has been an important vector of the alphavirus chikungunya in the 2004–2007 epidemic across several Indian Ocean islands [7], the 2007 concurrent outbreak with dengue in Gabon [8], and the 2007 and 2017 outbreaks in Italy [9,10]. Additionally, *Aedes albopictus* has also been implicated as a vector of other groups of arboviruses including the orthobunyavirus La Crosse virus [11], which is an enzootic peribunyaviridae involved in neurological disorders in North Carolina, where this study takes place [12]. *Aedes albopictus* control often relies on the use of adulticides [13], as is the case in North Carolina, where pyrethroid insecticides are commonly used for barrier spraying to control *Ae. albopictus* [14]. While insecticide resistance has been documented in *Ae. aegypti* populations around the world, fewer studies have focused on the resistance status of *Ae. albopictus*, with most work on this species concentrated in Southeast Asia, where resistance to all four major insecticide classes has been reported [5]. Previous work in the United States found that *Ae. albopictus* populations remained broadly susceptible to most insecticide treatments, though low levels of resistance to organophosphates and dichlorodiphenyltrichloroethane (DDT) have been detected in Florida and New Jersey populations [15]. However, more recently, 30% of *Ae. albopictus* populations collected throughout Florida were found to be resistant to pyrethroid insecticides [16], indicating that resistance may be increasing. 

*Ae. albopictus* abundance varies across space and time, influencing local pathogen transmission potential. Socioeconomic, landscape, and seasonal factors have been associated with *Ae. albopictus* abundance in many studies. Areas of low socioeconomic status (SES) often have a greater number of discarded containers for *Ae. albopictus* breeding [17], and pupae from *Aedes* species are more likely to be found in neighborhoods below median income [18]. This was demonstrated in recent research in Mecklenburg County, North Carolina, where the abundance of gravid *Aedes albopictus* was significantly higher in low-income neighborhoods [19]. This work further identified land cover factors associated with *Ae. albopictus* abundance including the percent of land covered by buildings, tree canopy, grass and shrubs, roads and railroads, and the overall diversity of land cover types in a 30-meter buffered area around sampled sites [19,20]. Additional studies have indicated that small patches of vegetation in urban areas such as parks, gardens, and playgrounds are often associated with high *Ae. albopictus* abundance [21] and that peaks in abundance often occur in late summer months in temperate climates [19,21,22].

This study aimed to establish a baseline description of the insecticide resistance status and patterns of morphological variation within a population of *Ae. albopictus* collected from Mecklenburg County, North Carolina. As such, our first objective was to screen adult *Ae. albopictus* females for genetic mutations indicating resistance to pyrethroids, the most commonly used class of insecticides for barrier spraying in North Carolina [14]. Additionally, we hypothesized that variation in female *Ae. albopictus* size would be associated with the socioeconomic, landscape, and seasonal factors that influence *Ae. albopictus* abundance. Previous studies have found that female *Ae. albopictus* size is positively correlated with fecundity [23,24]. We predicted that we would observe larger mean wing lengths in the lower socioeconomic classes, land cover types, and time periods associated with higher *Ae. albopictus* abundance. Furthermore, vector size influences virus transmission potential, with viral dissemination more likely among smaller individuals, as has been shown for dengue virus in *Ae. albopictus* [25] and La Crosse virus in *Ae. triseriatus* [26]. If size can serve as a reliable proxy for abundance or disease transmission potential in a local context, it provides a low-cost means to prioritize and target areas of importance, rather than time-consuming abundance sampling measures.

## 2. Materials and Methods

### 2.1. Study Site

**Figure 1:**
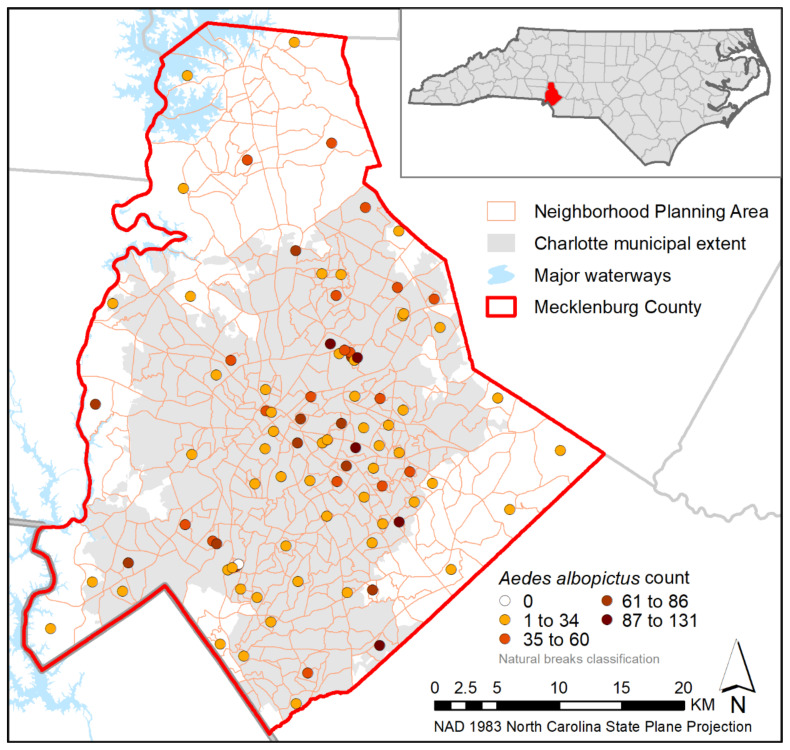
Collection sites in Mecklenburg County, North Carolina. Sites are colored to represent the total number of *Ae. albopictus* collected at that location during this study. Inset map shows location of Mecklenburg County in North Carolina.

We performed our analyses using adult female *Aedes albopictus* collected from June to August 2017 in Mecklenburg County, North Carolina, which encompasses the city of Charlotte (Figure 1). Mecklenburg County has an average population density of approximately 1900 people per square mile and a median household income of $61,695, with 13.4% of the population classified as persons in poverty in 2017, when these samples were collected [27], and the city of Charlotte has been characterized as having pervasive racial segregation and income inequality [28,29]. 

The *Ae. albopictus* specimens used in this study were collected from 90 unique sampling sites selected to maximize spatial distribution across the county and to represent the range of values present across a variety of socioeconomic and landscape factors [19,20]. Briefly, sampling was conducted using Gravid *Aedes* Traps (GATs) with hay-infused water as an attractant. Traps were emptied on a weekly basis for twelve weeks and specimens were identified morphologically when possible [30] or genetically verified to species at the Walter Reed Biosystematic Unit when specimens were degraded. The majority (72%) of collections included *Ae. albopictus*, with this species representing 86% of the total number of mosquitoes collected. Other identified species included *Ae. triseriatus*, *Ae. vexans*, *Ae. japonicus*, *Culex resuans*, and *Cx. Pipiens* [19]. 

### 2.2. DNA Extraction, Amplification, and Sequencing

We first aimed to determine whether the *Ae. albopictus* population in Mecklenburg County had any genetic mutations that would indicate resistance to pyrethroid insecticides. We therefore destructively extracted DNA from whole mosquitoes for use in polymerase chain reaction (PCR) using Qiagen DNeasy Isolation Kits (Qiagen Sciences, Germantown, MD, USA). For all samples, we amplified and sequenced two regions of *kdr* (domain II, 381 bp; domain IV, 280 bp) using the AegSCF20/AegSCR21 and AlbSCF6/AlbSCR8 primer pairs, respectively (Appendix A). Amplification of *kdr* domain III was unsuccessful. The thermocycler conditions were identical for *kdr* domains II and IV, an initial denaturing step at 96 °C for 10 min, 40 cycles of 30 s at 96 °C, 30 s at 55 °C, and 45 s at 72 °C, with a final extension step of 10 min at 73 °C (Appendix A, Table A1). All mosquito PCR products were cleaned using exonuclease I and shrimp alkaline phosphatase (Fisher Scientific, Pittsburgh, PA, USA). Primer extension sequencing was performed by Genewiz (South Plainfield, NJ, USA) using Applied Biosystems BigDye version 3.1. The reactions were then run on Applied Biosystem’s 3730xl DNA Analyzer. 

We used MegaX [31] and BioEdit [32] to assemble and form contigs of our forward and reverse reads.

### 2.3. Wing Length Measurements and Statistical Tests

We aimed to measure the wing length of one *Ae. albopictus* adult female from each of the 90 collection sites for each month in the collection window. However, because some sites did not yield *Ae. albopictus* females each month or specimens were in poor condition, we measured 236 wings total (representing 84, 72, and 80 sites in June, July, and August, respectively). We used a camera attached to a dissecting microscope to photograph the mosquito wings and then processed all images with ImageJ [33] to measure the length of each wing. Each wing was measured by two of the authors (S.M. and E.S.) independently. Measurements were averaged to determine a consensus length. If there was a difference greater than 2 mm between the independent measurements, a third measurement was taken by a third author (GH) and computed into the average for the month.

To test for statistically significant associations between socioeconomic variables and wing length, we first tested for wing length differences across socioeconomic quintiles based on the 2016 median household income at the neighborhood planning area (NPA) level. The NPA is a unit developed by the Charlotte-Mecklenburg Planning Commission that approximates the census tract, but with improved representation of actual neighborhoods within the county [19]. Mean wing length measurements across the sampling period per site were tested for normality through visual assessments of plotted distributions and the Shapiro–Wilk test for normality and found not to be normally distributed (*p* value < 0.004). Since the data remained abnormal even after modification, we conducted a Kruskal–Wallis test and a subsequent pairwise Wilcoxon rank sum test to identify statistically significant differences in wing length across income groups. We tested for associations between mean wing length and the socioeconomic or human demographic variables at the NPA level that have been shown to be related to *Ae. albopictus* abundance in the study area [19]. We used Spearman rank correlations due to the non-normal distributions of the explanatory and response variables. These variables included violent crime rate, population density, employment rate, proportion Hispanic population, foreclosure rate, proximity to a park, and proportion African-American population [19]. All statistical tests were performed using base functions in R v3.5.0 (R Core Team, 2019).

We used the 2012 Mecklenburg County Tree Canopy/Land Cover dataset to test for associations between land cover and wing length. This dataset was developed at a 3.33-foot spatial resolution using object-based image analysis techniques along with 2012 LiDAR data, 2012 National Agriculture Imagery Program imagery, and ancillary spatial datasets [34]. Land cover types included buildings, roads/railroads, tree canopy, grass/shrubs, water, and other paved surfaces. We generated a 30-meter buffer around each sampling site and calculated the percentage of each land cover type present within each buffer. Previous work has indicated that a 30-meter buffer is the best scale to detect the relationship between high-resolution land cover variables and *Aedes* abundance [35]. We tested for correlations between percent of each land cover type present within the buffer and mean wing length at each collection site using Spearman rank correlations. Additionally, we used a Kruskal–Wallis test to identify significant differences in mean wing length at sites classified as rural (*n* = 5), suburban (*n* = 63), and urban (*n* = 20) for each collection month and the total sampling season. These designations were based on percent impervious surface (roads/railroads, other paved surfaces, and buildings) within the 30-meter buffer, based on cut-off values used in similar research [36]. We tested for statistically significant differences in mean wing length across collection months using a Kruskal–Wallis test and post-hoc Wilcoxon rank sum tests for pairwise differences. We used Global Moran’s I tests to detect spatial autocorrelation in the mean wing lengths across the study area for each month and for the averaged wing lengths for the entire sampling period using the point locations of sampled sites as inputs and inverse distance to conceptualize spatial relationships. All spatial data processing was completed in ArcGIS 10.6 (ESRI, Redlands, CA, USA), a commercial geographic information system.

## 3. Results

We found no mutations that would infer pyrethroid resistance among our samples from Mecklenburg County, NC. We successfully extracted DNA from 86 mosquitoes, representing 95% of the total 90 collection sites. Amplification and sequencing of *kdr* domains II and IV were successful for 27 individuals (30% coverage) and 75 individuals (83% coverage), respectively. The resulting sequences for all samples were deposited in GenBank; accession numbers can be found in Appendix B, Table A2.

The mean wing length for the 236 female *Ae. albopictus* specimens was 2.73 mm (range 1.64 mm to 4.29 mm). The results from the Kruskal–Wallis test to identify differences in wing length across median income quintiles were not statistically significant (Kruskal–Wallis χ^2^ = 2.645, df = 4, *p* = 0.619). The Spearman rank correlations between the socioeconomic and human demographic variables identified as being associated with *Ae. albopictus* abundance and mean wing length did not yield statistically significant associations [19]. 

Tests for associations between land cover and wing length did not yield statistically significant results. This included the Spearman’s rank correlation between the percentage of each land cover type present within the 30-meter buffer around each sampling site and the mean wing length at that site. The Kruskal–Wallis test for differences in mean wing length across rural, suburban, and urban areas based on percent impervious surface did not yield statistically significant results (Kruskal–Wallis χ^2^ = 1.275, df = 2, *p*-value = 0.529).

**Figure 2:**
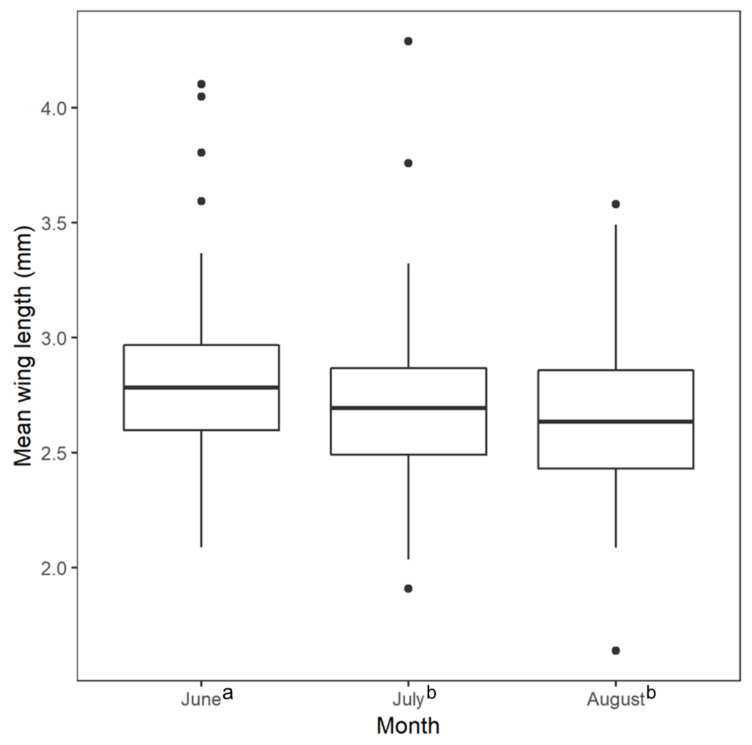
Mean female *Aedes albopictus* wing length by collection month. The mean June wing length was longer than the July and August mean wing lengths. There was no significant difference between the July and August mean wing length. Superscript lowercase letters indicate values significantly different from one another in the Kruskal–Wallis tests with a post-hoc Wilcoxon rank sum test at *p* ≤ 0.05.

The result from the Kruskal–Wallis test for differences in mean wing length across the three sampling months was statistically significant (Figure 2; Kruskal–Wallis χ^2^ = 9.950, df = 2, *p*-value = 0.007). We found a statistically significant difference between wing length measurements of samples collected in June and August (Wilcoxon rank sum test, *p*-value = 0.008) and between June and July (Wilcoxon rank sum test, *p*-value = 0.022), but not July and August (Wilcoxon rank sum test, *p*-value = 0.540). The mean wing lengths of collected mosquitoes was longest in June. The Global Moran’s I tests for spatial autocorrelation in mean wing lengths did not show statistically significant clustering or dispersal when averaged over the entire study period (*p*-value = 0.668) and for each month individually (June *p*-value = 0.983; July *p*-value = 0.279; August *p*-value = 0.738).

## 4. Discussion

As the range of *Ae. albopictus* continues to expand, continuous surveillance and study of the species is needed. Regular monitoring of insecticide susceptibility is essential to promptly identify the emergence of resistance and implement appropriate and alternative control measures [37]. Similarly, having a baseline understanding of the morphology and distribution of vector populations within the context of local socioeconomics, landscape, and temporal influences can inform targeted abatement strategies. In this study, we screened *Ae. albopictus* collected from Mecklenburg County, North Carolina, for genetic indicators of resistance and examined spatial and temporal patterns of wing length variation among the collected adult female *Ae. albopictus* specimens.

While the sample size of specimens that we were able to successfully extract and amplify genetic material from was relatively small, which is a limitation, the homogenous lack of voltage-gated sodium channel mutations across the study area strongly suggests that this population is broadly susceptible to pyrethroid insecticides. This matches findings from similar studies in the area. In a 2018 study, researchers found that *Ae. albopictus* populations from seven North Carolina counties including Mecklenburg County were susceptible to five commonly used pyrethoids in CDC bottle bioassays, with the exception of Pitt County, where developing resistance (93% mortality) to permethrin was documented [14]. In contrast, resistance to chlorpyrifos and malathion, two commonly used organophosphates, was documented in all seven populations in the same study [14]. Budgets for mosquito control programs in North Carolina have been dramatically reduced in the past decade [38] and a recent survey found that approximately 31% of respondents in North Carolina personally administered insecticides for mosquito control on their property [39]. This combination of limited resources for oversight and unregulated insecticide applications by private individuals indicates that selection for insecticide resistant mosquitoes will likely continue in this area, although more information is needed to predict whether pyrethroid resistance will develop.

We did not detect significant associations between *Ae. albopictus* wing length and most of the socioeconomic and landscape factors considered in this study, although these factors were associated with *Ae. albopictus* abundance in previous research. This means that our hypothesis that larger female *Ae. albopictus* females with higher fecundity drive increases in local abundance was not supported. While certain areas may produce larger female *Ae. albopictus* that have more offspring, their impact on local abundance could be countered by high larval densities that result in smaller adults [40]. Furthermore, the results from the Global Moran’s I tests indicate that wing length did not exhibit spatial autocorrelation, suggesting that female adult size is likely to be the result of multiple random, interacting processes. We did not observe significant differences in wing length between rural, suburban, and urban sites. This is contradictory to recent work conducted in Athens, Georgia, that found that *Ae. albopictus* emerging from containers placed in urban sites were significantly smaller than those placed in rural sites [36]. However, this difference was statistically significant only in the fall, and our study was limited to a single summer season of collections. Differences in wing length across land cover types could increase if sampling in Mecklenburg County were to continue into the fall.

We found that *Ae. albopictus* collected in June had significantly longer wing lengths than *Ae. albopictus* collected in August and July, while the average number of *Ae. albopictus* collected in the study area was the lowest in June [19], indicating the larger wing spans were not associated with greater abundance. This difference in size could be due to meteorological conditions. The total monthly amounts of precipitation for Charlotte in June, July, and August of 2017 were 4.3 inches, 4.45 inches, and 5.29 inches, respectively [41]. Observing significantly larger mosquitoes during the month with the least amount of rainfall corresponded with a previous study that found that *Ae. albopictus* reached their largest size under conditions where their water source was allowed to evaporate completely. In this scenario, increased mortality during the aquatic life stages resulted in fewer adults emerging, but the surviving individuals were larger, possibly due to decreased competition for resources [42]. Additionally, temperature conditions were lower in June than in July or August during the study period, with an average high of 29.8 ℃ in June compared to 33.1 °C in July and 30.8 °C in August. Higher temperatures have been found to result in the growth of heavier adult *Ae. albopictus* with shorter wings [43]. Further work would likely determine the extent to which these meteorological variables interact with each other and other environmental factors to determine *Ae. albopictus* size.

## 5. Conclusions

In conclusion, this work served to establish a baseline description of the *Ae. albopictus* population in Mecklenburg County, North Carolina. While genetic indicators of pyrethroid resistance were not detected, continued surveillance remains critical for early detection of diminished susceptibility. Additionally, while we did not see significant associations between *Ae. albopictus* wing lengths and several factors that have been linked to abundance in this and similar species, we did observe temporal variation in the wing lengths of this population. Further research will likely illuminate the extent to which spatial and temporal factors influence variation in wing size and other morphological traits in *Ae. albopictus* and other mosquito species. 

## Data Availability

The data used to support the findings of this study are included within the article.

## Appendix A

**Table A1:** Primers Used for Amplification of Three Domains of *kdr*.

kdr domain II	Forward	5’-GACAATGTGGATCGCTTCCC-3’	Kasai et al., 2011 [44]
	Reverse	5’-GCAATCTGGCTTGTTAACTTG-3’	
kdr domain III	Forward	5’-GAGAACTCGCCGATGAACTT-3’	Kasai et al., 2011 [44]
	Reverse	5’-AACAGCAGGATCATGCTCTG-3’	
kdr domain IV	Forward	5’-TCGAGAAGTACTTCGTGTCG-3’	Kasai et al., 2011 [44]
	Reverse	5’-AACAGCAGGATCATGCTCTG-3’	

## Appendix B

**Table A2:** Accession Numbers for *kdr* Domains II and IV.

Site ID	Neighborhood Classification	*kdr* Domain II	*kdr* Domain IV
2	4		MZ964062
3	1		MZ964063
4	2		MZ964064
5	3		MZ964065
6	1		MZ964066
7	2		MZ964067
8	1		MZ964068
9	1		MZ964069
10	4		MZ964070
11	2	MZ964035	MZ964071
12	3		MZ964072
13	4		MZ964073
14	5		MZ964074
16	2	MZ964036	MZ964075
17	3	MZ964037	MZ964076
18	4		MZ964077
19	5		MZ964078
20	1	MZ964038	MZ964079
22	3		MZ964080
23	5	MZ964039	MZ964081
24	5	MZ964040	MZ964082
25	1		MZ964083
26	4		MZ964084
27	3	MZ964041	MZ964085
28	2	MZ964057	MZ964086
29	4		MZ964087
30	2	MZ964058	MZ964088
31	3		MZ964089
33	3	MZ964042	MZ964090
34	4		MZ964091
35	3		MZ964092
37	1		MZ964093
38	1		MZ964094
40	4	MZ964043	
41	5	MZ964056	MZ964095
42	4	MZ964044	MZ964096
43	5		MZ964097
44	3	MZ964045	MZ964098
45	4		MZ964099
47	3		MZ964100
48	3		MZ964101
50	5	MZ964046	MZ964102
51	5	MZ964047	
52	5		MZ964103
53	1	MZ964048	MZ964104
54	3	MZ964049	MZ964105
55	4		MZ964106
56	3		MZ964107
57	2	MZ964050	MZ964108
58	4		MZ964109
59	4		MZ964110
61	5	MZ964051	MZ964111
62	2	MZ964052	MZ964112
63	1		MZ964113
64	3		MZ964114
65	2		MZ964115
66	2	MZ964053	MZ964116
67	3		MZ964117
68	5		MZ964118
69	2	MZ964054	MZ964119
71	1		MZ964120
72	5		MZ964121
73	2		MZ964122
75	3		MZ964123
76	1		MZ964124
77	4		MZ964125
78	1	MZ964055	MZ964126
79	5		MZ964127
80	4	MZ964059	MZ964128
81	4		MZ964129
82	5		MZ964130
83	1		MZ964131
85	1		MZ964132
86	5		MZ964133
87	4		MZ964134
88	1	MZ964060	
89	5	MZ964061	MZ964135
